# Multi-organ frailty is enhanced by periodontitis-induced inflammaging

**DOI:** 10.1186/s41232-025-00366-5

**Published:** 2025-02-03

**Authors:** Yoshitaka Kase, Satoru Morikawa, Yuji Okano, Tatsuya Hosoi, Takazumi Yasui, Yoko Taki-Miyashita, Mitsutaka Yakabe, Maraku Goto, Kazuyuki Ishihara, Sumito Ogawa, Taneaki Nakagawa, Hideyuki Okano

**Affiliations:** 1 Regenerative Medicine Research Center, Keio University, 3-25-10 Tonomachi, Kawasaki-Ku, Kawasaki-Shi, 210-0821 Japan; 2 Division of CNS Regeneration and Drug Discovery, International Center for Brain Science (ICBS), Fujita Health University, 1-98 Dengakugakubo, Kutsukake-Cho, Toyoake-Shi, Aichi, 470-1192 Japan; 3 Department of Dentistry and Oral Surgery, Keio University School of Medicine, 35 Shinanomachi, Shinjuku-Ku, Tokyo, 160-8582 Japan; 4 Department of Geriatric Medicine, Graduate School of Medicine, The University of Tokyo, 7-3-1 Hongo, Bunkyo-Ku, Tokyo, 113-8655 Japan; 5 Department of Extended Intelligence for Medicine, The Ishii-Ishibashi Laboratory, Keio University School of Medicine, 35Shinjuku-Ku, ShinanomachiTokyo, 160-8582 Japan; 6 Department of Microbiology, Tokyo Dental College, 2-1-14 Kanda-Misaki-Cho, Chiyoda-Ku, Tokyo, Japan; 7 Oral Health Science Center, Tokyo Dental College, 2-9-18, Kanda-Misaki-Cho, Chiyodaku, Tokyo, Japan

**Keywords:** Inflammaging, Periodontitis, Frailty, Osteopenia, Cognitive decline, Cognitive frailty

## Abstract

**Background:**

The incidence of periodontitis is high in older individuals. However, its impact on multi-organ frailty remains unclear. We developed mouse models with varying severity and duration of periodontitis to examine its effects.

**Methods:**

We generated mouse models with mild and severe periodontitis, categorizing the disease duration into 3-month and 5-month periods for analysis. The organs assessed for frailty included the gastrocnemius muscle, soleus muscle, brain, and femur.

**Results:**

Our study found that periodontitis induced systemic inflammation resembling inflammaging and other symptoms characteristic of age-induced frailty. Notably, muscle impairment developed specifically in slow-twitch muscles, and the femur emerged as the most vulnerable bone, exhibiting reduced bone mineral density even with mild and short-duration periodontitis. This condition resulted in the co-occurrence of bone fragility and slow-twitch muscle dysfunction. Cognitive function assessment revealed increased activated microglia and decreased adult neurogenesis in the hippocampus, impairing spatial learning. Thus, periodontitis induced both physical and cognitive frailties. Therapeutic intervention for the periodontitis, which halted the exacerbation of bone resorption markers, did not restore femur bone mineral density.

**Conclusion:**

This study underscores the role of periodontitis in inducing multifaceted organ frailty with vulnerability, varying by organ, and the necessity of early intervention, particularly regarding bone density loss.

**Supplementary Information:**

The online version contains supplementary material available at 10.1186/s41232-025-00366-5.

## Background

The incidence of frailty is rising alongside the global increase in life expectancy. Frailty is the increased risk of adverse health outcomes due to the functional decline of multiple organs in older individuals [1]. It encompasses physical weakness, functional decline, cognitive decline, and psychological vulnerability [2], significantly impacting both life expectancy and quality of life [1].

Sarcopenia and dynapenia are common in older individuals. Sarcopenia is a decline in muscle mass and strength, leading to reduced physical function and an increased risk of falls [3, 4]. Dynapenia involves fatty degeneration and loss of muscle function, while the muscle mass remains unchanged [5]. Dynapenia may precede sarcopenia [4]. Both conditions significantly impact the activity levels and hospitalization rates of older individuals [1]. Therefore, from a healthcare cost perspective, effective prevention and management are crucial.

Frailty can be triggered and exacerbated by aging, inflammation, and disease [6]. Hospitalization due to falls or fractures can decrease physical activity, resulting in dynapenia and sarcopenia. Recently, the concept of “inflammaging” has been proposed, highlighting how aging is associated with a chronic, systemic, low-grade inflammatory state, which can be enhanced by diseases and other factors and accelerates frailty [7, 8].

However, levels and areas of inflammaging and frailty differ between individuals. Research investigating how tissue-specific low-grade chronic inflammations, such as periodontitis, can affect multiple organs and lead to varying degrees of frailty is insufficient. Periodontitis is an oral inflammatory disease prevalent in older individuals but is often neglected in routine medical care and daily life because it is perceived as less severe than other—often life-threatening—inflammatory diseases, such as pneumonia and bedsores [9].

Despite this neglect, a strong association of periodontitis with age-related alveolar bone loss and an adverse impact on systemic health is seen [10, 11]. Therefore, we used mouse models to experimentally investigate the causal relationship between periodontitis and frailty, addressing the growing interest in its impact on various organs. While clinical observations and retrospective studies have suggested a link between periodontitis and frailty, the direct effects of periodontitis on multiple organs have not been thoroughly explored. Consequently, although oral care is important to prevent periodontitis in geriatrics, its significance and priority in preventing frailty among older individuals have remained unclear. This reverse-translational research is thus urgently needed, as the effects of periodontitis on various organs are increasingly gaining attention.

Periodontitis and cognitive impairment and/or dementia appear closely linked. A cohort study found that the number of teeth affected by periodontitis, rather than the total number of remaining teeth, was linked to hippocampal atrophy in older individuals [12]. Simply having many remaining teeth is not associated with protection, as a high number of teeth with periodontitis can worsen cognitive function. The presence of the periodontitis-causing pathogen *Porphyromonas gingivalis* (Pg) and its toxic gingipain proteases in the brain of patients with Alzheimer’s disease (AD) suggests a potential role of Pg in AD pathogenesis [13, 14]. Another study indicated that intraperitoneal administration of Pg to mice increased amyloid-beta (Aβ) production in their brains, although this was observed in a mouse model of systemic Pg administration [15]. Despite these intriguing findings, it remains unclear in geriatric care whether cognitive decline leads to a breakdown in oral care that results in periodontitis or if periodontitis directly causes cognitive decline. The potential role of periodontitis in the development of cognitive frailty, a combination of cognitive decline and physical frailty, has not yet been conclusively demonstrated.

Regarding skeletal muscle, research has indicated that Pg infection can contribute to metabolic syndrome and adiposity in skeletal muscle [16]. However, the specific impact of the duration and severity of periodontitis on muscle health remains largely unexplored.

Similarly, the influence of periodontitis on bone health is not well understood. While numerous studies have examined its effects on alveolar bone [17, 18], research investigating its impact on the femur is lacking. While the influence of periodontitis on the alveolar bone, mediated by increased receptor activator of nuclear factor-κB ligand (RANKL) expression, has been extensively studied [9, 19], its effects on the femur, a critical factor in frailty, remain poorly understood. It is unclear whether periodontitis contributes to femoral osteopenia or if the femur compared with other organs is more susceptible to frailty, based on disease severity and duration.

To address these knowledge gaps, we developed mouse models of periodontitis with varying severity and duration to investigate the impact of chronic periodontitis-induced inflammation on the muscles, brain, and femur and elucidate the progression of inflammation, a process linking chronic inflammation to accelerated aging. Our findings underscore the importance of preventing and treating periodontitis, which is often overlooked owing to its low-grade inflammatory nature. Furthermore, we provide a scientific discourse on the effects of systemic inflammation and aging, emphasizing the interconnectedness of multiple organs in the body rather than focusing solely on individual organs. Clarifying the extent to which periodontitis causes frailty is of great significance for future dental and geriatric practice, not only as a model for chronic low-grade inflammation but also for understanding the far-reaching consequences of oral health on age-related health outcomes.

## Results

### Confirmation of alveolar bone resorption in a mouse model of periodontitis

**Fig. 1:**
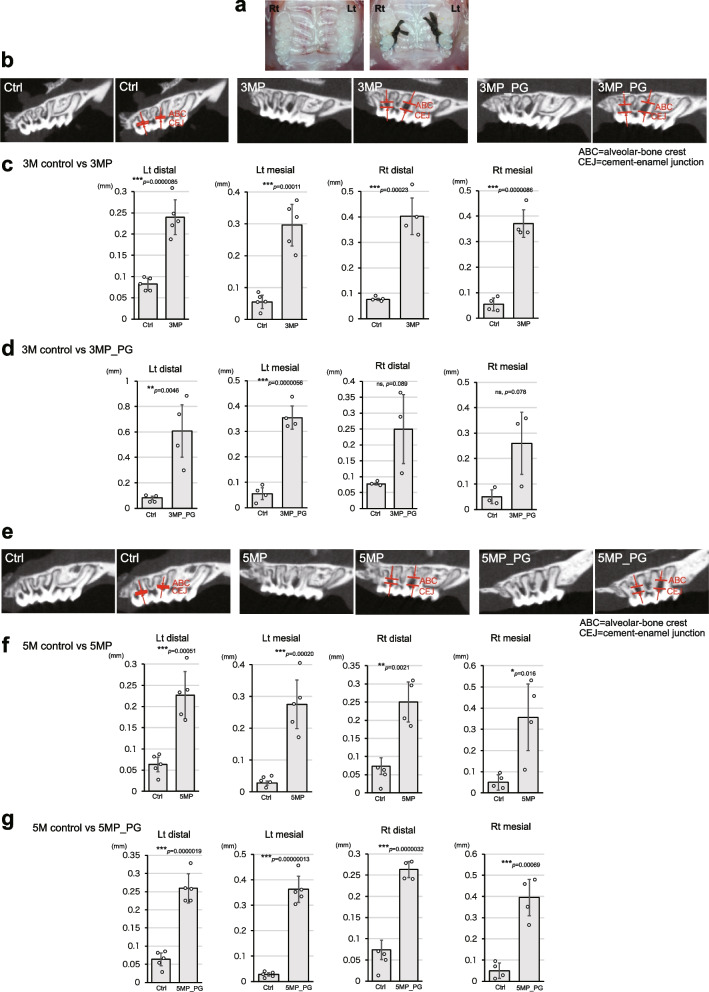
Silk thread ligation induces periodontitis, leading to alveolar bone resorption. **a** Representative images showing silk threads ligated around the maxillary second molars of a mouse to induce periodontitis. **b**–**g** Assessment of alveolar bone resorption in the 3-month (**b**–**d**) and 5-month (**e**–**g**) period periodontitis models without and with Pg, compared with controls (Ctrl), using CT imaging. **b** CT images of the left second molars from Ctrl, 3MP, and 3MP_PG groups. **c**, **d** Quantification of the distance between the alveolar bone crest (ABC) and cemento-enamel junction (CEJ) at the distal and mesial sites of the second molars. **e** CT images of the left second molars from Ctrl, 5MP, and 5MP_PG groups. **f**, **g** Quantification of ABC-CEJ distance at the distal and mesial sites of the second molars. Five mice (*n* = 5) were used for the analysis in 3MP of the left site and 5MP of the left site and 5MP_PG of the left site. Four mice (*n* = 4) were used for the analysis in 3MP of the right site and 3MP_PG of the left site and 5MP of the right site and 5MP_PG of the right site. Three mice (*n* = 3) were used for the 3MP_PG of the right site. Data are presented as mean ± SE. Statistical significance was determined using an unpaired two-tailed Student’s *t*-test

We developed mouse periodontitis models with varying disease severities and durations to investigate the impact of the disease on various organs. The first model, termed 3MP (3 months, periodontitis), involved ligating the maxillary second molars bilaterally with silk threads for 3 months (Fig. 1a), representing sustained periodontitis for this duration. In the 3MP_PG model, in addition to silk thread ligation, mice were administered Pg, a keystone bacterium known to disrupt commensal flora and cause periodontitis [20–22], into the oral cavity using a methylcellulose solution three times a week for 3 months. This approach represented periodontitis. The 5-month variations of these silk thread ligation models, without or with Pg, were designated 5MP and 5MP_PG, respectively.

To confirm the presence of periodontitis, alveolar bone resorption was quantified. In the 3-month models, the distance between the alveolar bone crest (ABC) and cementoenamel junction (CEJ) was significantly increased in both the 3MP and 3MP_PG groups compared with the control (3M_Ctrl) group (Fig. 1, b–d). A similar pattern was observed in the 5-month models, with an increased ABC-CEJ distance in both the 5MP and 5MP_PG groups compared with the 5 M Ctrl group (Fig. 1, e–g). This increased distance suggests ongoing bone resorption, confirming that our models (3MP, 3MP_PG, 5MP, and 5MP_PG) successfully induced alveolar bone resorption, as reported previously. Alveolar bone resorption appeared already maximized within the 3MP model, as no further increases were observed when the disease period extended to 5 months and/or Pg was administered. However, the phenotypes and degrees of systemic inflammation significantly differed between the four periodontitis models, as discussed below.

### Periodontitis specifically disrupts slow-twitch muscles

We initially investigated the impact of periodontitis on skeletal muscle. This analysis aimed to explore the effects of periodontitis on fast and slow muscle fibers in terms of gene expressions to help explain the changes in macro phenotypes. To achieve this, we conducted a comprehensive examination of the gastrocnemius muscles from 5M_Ctrl, 5MP, and 5MP_PG mice, utilizing single-cell RNA sequencing (scRNA-seq). After data preprocessing, we performed factor analysis to extract both fast twitch fibers and slow twitch fibers from heterogeneous samples. We selected a combination of marker genes for fast (Myh1, Myh2, and Myh4) and slow (Myh7) twitch, as well as other similar genes (Myh7b, Myh11, and Myh3), based on GO terms (Supplementary Fig. 1a), and utilized them for factor analysis aiming to represent the authenticity of both fast twitch and slow twitch as factor scores. Leveraging the same workflow introduced in our previous study [23] (Supplementary Fig. 1, b-f), we identified two factors: factor 1, which represented fast-twitch fibers, and factor 2, which corresponded to slow-twitch fibers (Supplementary Fig. 1e). Using manually determined thresholds referring to histograms of the factor scores (Supplementary Fig. 1f), we classified the samples into four groups: double-negative, fast-twitch, slow-twitch, and double-positive (Supplementary Fig. 1 g). The sample conditions (i.e., 5M_Ctrl, 5MP, and 5MP_PG) seemed irrelevant to factor score coordinates (Supplementary Fig. 1 h) or class balances of the four categories (Supplementary Fig. 1i). Further analyses were conducted on the fast-twitch (Supplementary Fig. 1j) and slow-twitch (Supplementary Fig. 1 k) subgroups using the stratification defined in this study.

**Fig. 2:**
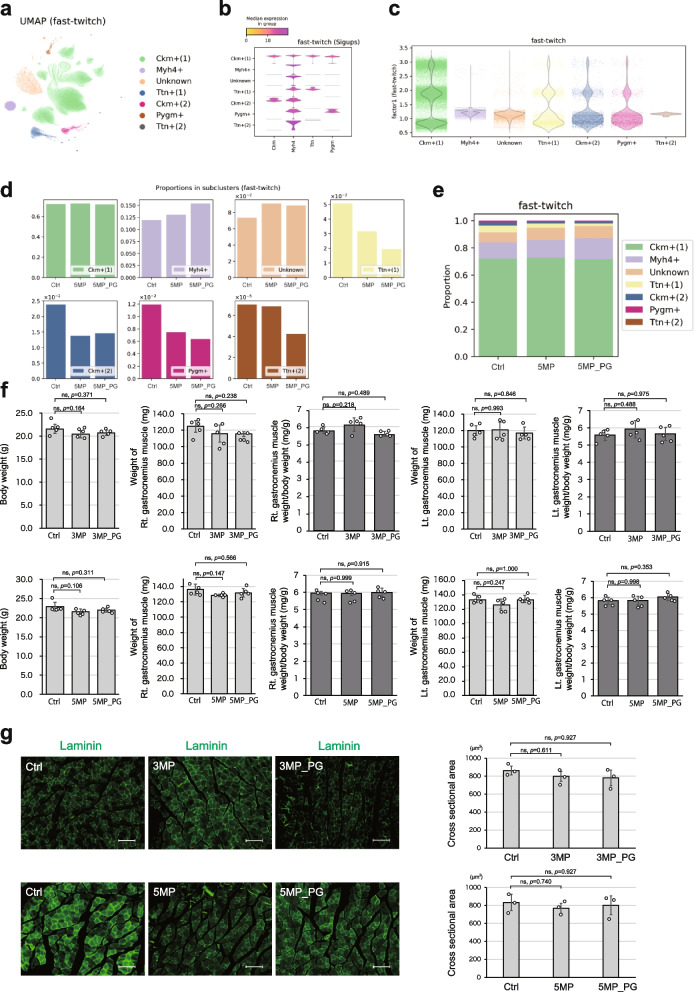
Periodontitis did not significantly impair fast-twitch muscles. **a** Scatter plot of fast-twitch samples colored by clusters in UMAP embedding. Clusters were named after their significantly upregulated genes (sigups). If several clusters shared the same sigups, their names were indexed. If no applicable sigups were found, the cluster was named Unknown. **b** Violin plots of the sigups for the clusters. **c** Violin plots of the factor 1 scores (corresponding to the likelihood of being fast-twitch) by clusters. Raw data are also overlaid with horizontal jitters. **d**, **e** Sample sizes of the clusters stratified by experimental conditions. **f** Quantitative graphs depicting body weight, right and left gastrocnemius muscle weight, and right and left gastrocnemius muscle/body weight ratio of mice in each group (*n* = 5). **g** Immunostained image of gastrocnemius muscle using an anti-laminin antibody on a formalin-fixed paraffin-embedded (FFPE) sample, along with quantification of the cross-sectional area (*n* = 3). The upper panel displays the 3-month disease model, while the lower panel shows the 5-month disease model. Data are presented as mean ± SD. One-way ANOVA followed by the Tukey–Kramer test was applied for group analysis. *p < 0.05, ns: not significant. Scale bar: 100 μm

To evaluate disease-related transcriptomic variations, we identified seven clusters in the fast-twitch subpopulation and annotated them referring to their differentially expressed genes (Fig. 2a, b). To profile their characteristics, we visualized the distribution of the factor 1 scores of each class. The cluster named Ckm + (1) had the largest population of high scores, indicating that Ckm + (1) represents the most typical population of fast-twitch fibers (Fig. 2c). To understand the collective features of the gastrocnemius muscle samples, we tallied the class balance proportions per condition (Fig. 2d, e). The Ckm + (1) cluster was consistently dominant across different severities of the periodontitis models, suggesting that a major part of the fast-twitch fibers was not affected by the disease. However, variations associated with increased severity included an increase in the Myh4 + cluster and decreases in the Pygm + , Ttn + (1) and Ttn + (2) clusters. Taken together, changes in the relative abundance of the major population of fast-twitch fibers were not detected, whereas some minor subpopulations were affected by the severity of the periodontitis models.

In accordance with the above data suggesting no reduction in the major population of fast-twitch fibers, there was no observable change in the body weight of mice between the 3-month (3M_Ctrl, 3MP, and 3MP_PG) and 5-month models (5M_Ctrl, 5MP, and 5MP_PG) (Fig. 2f), and to gauge potential atrophy in the gastrocnemius muscle, which primarily consists of fast-twitch muscle [24, 25], we compared the muscle weight ratio to the body weight of each mouse but found no decline in muscle weight (Fig. 2f). Additionally, despite including muscle atrophy markers such as Fbxo32 (atrogin-1) and Trim63 (murf-1) in our analysis, scRNA-seq of the gastrocnemius muscle revealed no significant alterations (Supplementary Fig. 2a, b). We also examined the cross-sectional area of the gastrocnemius tissue stained with laminin, observing no increase or decrease between the control group and the 3MP, 3MP_PG, and 5MP, 5MP_PG groups (Fig. 2g).

**Fig. 3:**
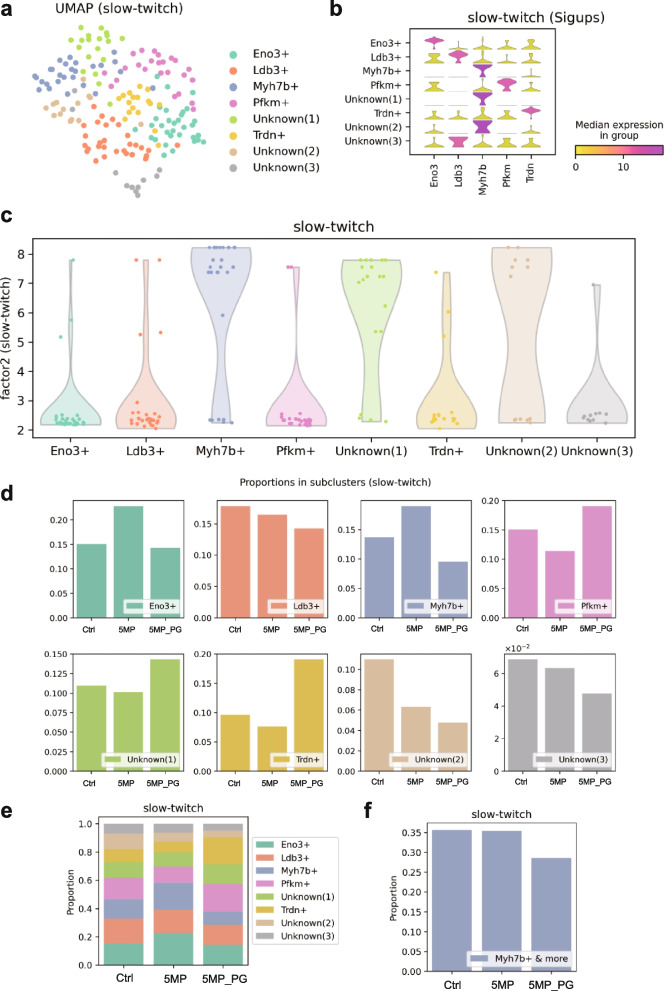
Periodontitis specifically impairs slow-twitch muscles. **a** Scatter plot of slow-twitch samples colored by clusters in UMAP embedding. The clusters were named after their significantly upregulated genes (sigups). Clusters without any applicable sigups were named Unknown, and those sharing the same sigups were indexed. **b** Violin plots of the sigups for the clusters. **c** Violin plots of the factor 2 scores (which corresponded to the likelihood of being slow twitch) by clusters. Raw data are overlaid with horizontal jitters. **d**, **e** Sample sizes of the clusters stratified by experimental conditions. **f** Combined cluster sizes of Myh7b + , Unknown(1), and Unknown(2) under respective conditions

Next, we performed the same set of the scRNA-seq analyses on the slow-twitch subpopulation and identified eight clusters named after their differentially expressed genes (Fig. 3a, b). Unknown(1) and Unknown(2) had no significant marker genes, but their gene expression patterns resembled that of Myh7b + (Fig. 3b). The distributions of the factor 2 scores (for slow-twitch likelihood) of these three clusters were also similarly high and much higher than of the other five clusters (Fig. 3c), prompting us to consider these three populations a representing typical characteristics of slow twitch fibers. In addition to the bar charts of the class balance proportion per experimental condition (Fig. 3d, e), we analyzed the same attribute for the combined Myh7b + , Unknown(1), and Unknown(2) fractions (Fig. 3f). This integrated population showed a decrease in the 5MP_PG mice compared to 5M_Ctrl and 5MP mice, suggesting that periodontitis triggered a reduction in the largest population with slow-twitch-specific characteristics.

**Fig. 4:**
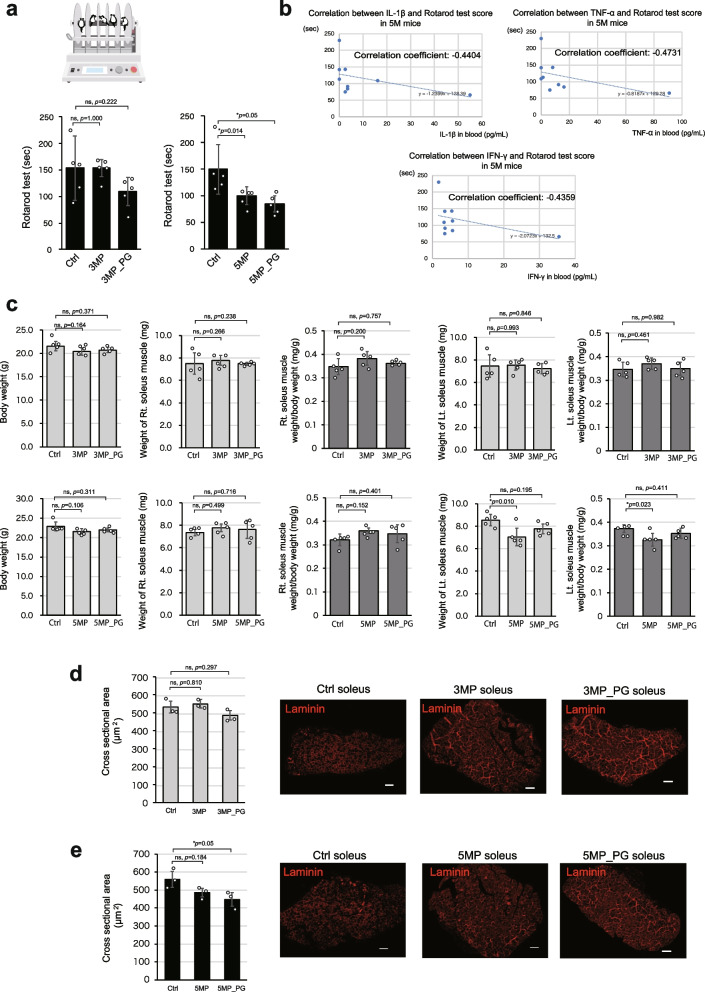
Periodontitis impairs slow-twitch muscles and leads to dynapenia. **a** Muscle performance was assessed using the rotarod test. The graph on the left illustrates results from the 3 M model, while the graph on the right depicts those from the 5 M model mice (*n* = 5). **b** Correlations of the serum levels of IL-1β, TNF-α, and IFN-γ with rotarod test scores. The rotarod performance negatively correlates with the levels of these inflammatory cytokines (*n* = 3). **c** Graphs depicting the body weight, right and left soleus muscle weight, and right and left soleus muscle/body weight ratio of mice in each group (*n* = 5). **d**, **e** Graphs of the quantified cross-sectional area of the soleus muscle, accompanied by representative micrographs of tissue sections immunostained with anti-laminin, in 3 M and 5 M-models, respectively (*n* = 3). Data are presented as mean ± SD. One-way ANOVA followed by the Tukey–Kramer test was applied for group analysis. *p < 0.05, ns: not significant. Scale bar: 100 μm

Based on the insights from our scRNA-seq analyses, indicating a specific impairment of slow-twitch muscles due to periodontitis, we conducted further assessments of muscle endurance and fatigue in model mice using the rotarod test, in which slow-twitch muscles play an important role (Fig. 4a). We observed decreased performance in 3MP_PG mice, although the difference compared to 3M_Ctrl and 3MP mice was not statistically significant (Fig. 4a, left graph). However, in the 5MP and 5MP_PG mice, there were significant declines in performance compared to the performance of 5M_Ctrl mice (Fig. 4a, right graph). The combined 3 M and 5 M data conclude that periodontitis negatively impacts rotarod test performance, with both the duration and severity of the disease probably playing a role.

Additionally, we quantified inflammatory cytokines IL-1β, TNF-α, and TNF-γ [6, 10] through blood sampling, finding an inverse correlation between the results of the rotarod test and increased levels of these cytokines (Fig. 4b). Because our scRNA-seq findings implied that periodontitis specifically affects slow muscle fibers, we extended our analysis to the soleus muscle, which, unlike the gastrocnemius muscle, is predominantly composed of slow-twitch fibers [24, 25].

Soleus muscle weight did not change significantly with periodontitis (Fig. 4c) as observed for the gastrocnemius muscle. However, laminin staining of soleus muscle tissue to assess the cross-sectional area of each sample showed decreases in mean myofiber area in the 3MP_PG (Fig. 4d), 5MP, and 5M_PG groups (Fig. 4e), with the latter two observations reaching statistical significance. The similarity in periodontitis-associated patterns between rotarod performance (Fig. 4a) and soleus muscle tissue cross-sectional areas (Fig. 4d, e) is notable. We assume this represents reductions in motor function through declines in slow-twitch muscle tissue integrity, induced by enhanced systemic inflammatory status resulting from periodontitis and affected by its duration and severity. In essence, while periodontitis appears not to directly affect muscle weight, it induces a deterioration in the quality of slow muscles, leading to susceptibility to fatigue.

### Periodontitis reduces bone mineral density in the femur

**Fig. 5:**
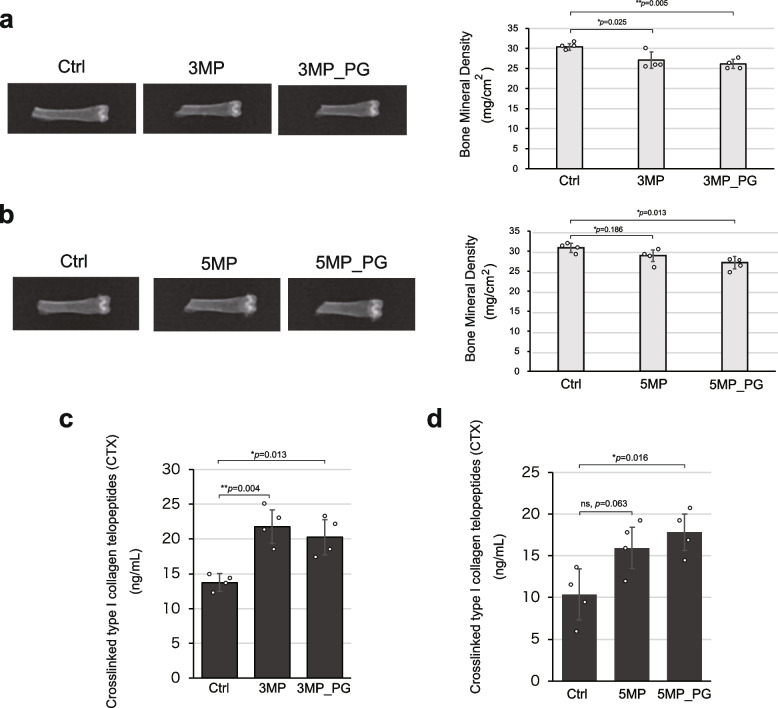
Periodontitis reduces the bone mineral density (BMD) in the femur. **a** Representative DXA images of femurs of the 3 M model mice, along with a quantitative graph of BMD. **b** Similarly, DXA images and a BMD graph for the 5 M model mice. **c**, **d** Graphs showing the quantification of the levels of serum C-terminal telopeptide of type I collagen (CTX) in serum, a bone resorption marker, in 3 M and 5 M model mice, respectively. Data are presented as mean ± SD. One-way ANOVA followed by the Tukey–Kramer test was applied for group analysis. *p < 0.05, **p < 0.01, (*n* = 4). ns: not significant

Next, we investigated how periodontitis affects the femur. The femur of each mouse model was analyzed by Dual-energy X-ray absorptiometry (DXA) [26]. The results showed that, unlike the muscle results, periodontitis significantly reduced bone mineral density even during the shortest disease period of 3 months (Fig. 5a, b). Bone mineral density was significantly decreased even after only 3 months of disease, indicating that this organ is susceptible to periodontitis.

We also measured bone metabolic markers. The levels of serum C-terminal telopeptide of type I collagen (CTX) [27, 28] was significantly increased by periodontitis in the 3 M and 5 M models (Fig. 5c, d). CTX is a marker of bone resorption released when bone matrix is degraded, suggesting increased osteoclast activity due to periodontitis. TRACP-5b [27, 28], another marker of bone resorption, also showed increased mean values in the periodontally affected groups, although the differences were not significant (Supplementary Fig. 3a).

Periodontitis in our models caused a decrease in bone density and osteopenia [26], in addition to the slow-twitch-specific muscle impairment described above.

### Periodontitis causes cognitive frailty by impairing spatial cognition

**Fig. 6:**
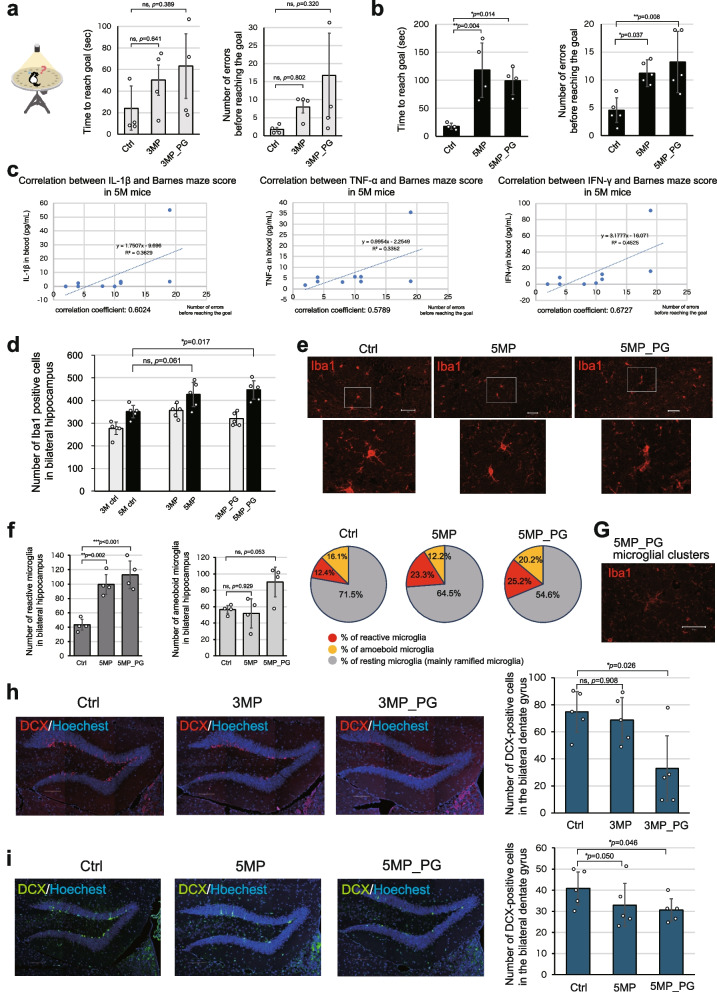
Periodontitis impairs spatial memory and affects cell functions in the brain. **a**, **b** Performances in the Barnes maze of the 3 M and 5 M model mice groups, respectively, were assessed by measuring the time taken to reach the goal and the number of incorrect turns made (*n* = 4). Periodontitis caused poorer performance. **c** Graph showing the correlation between serum IL-1β, TNF-α, and IFN-γ levels and Barnes maze performance in the 5 M groups. Poor performance is positively correlated with higher levels of these inflammatory cytokines (*n* = 3). **d** A graph quantifying Iba1-positive microglia in bilateral hippocampi of both the 3 M and 5 M groups (*n* = 5). The number of microglia significantly increased with disease severity in the 5-month disease model. **e** Representative micrographs of anti-Iba stained microglia in the hippocampus, shown together with magnified images. Scale bar: 50 μm. **f** Graphs and matching pie charts, quantifying the number of reactive microglia and amoeboid microglia in bilateral hippocampi and the proportion of microglia by morphology in the 5 M groups (*n* = 4). Periodontitis increased the number of reactive microglia. **g** Clusters of microglia in the hippocampus were observed only in the 5MP_PG group (*n* = 5). Anti-Iba stained tissue section; Scale bar: 50 μm. **h**, **i** Anti-DCX stained cells, indicative of newborn neurons, in the hippocampal dentate gyrus of 3 M and 5 M groups, respectively. Periodontitis decreased the number of DCX-positive cells. Scale bar: 100 μm. Data are presented as mean ± SD. Statistical significance was determined using an unpaired two-tailed Student’s *t*-test. One-way ANOVA followed by the Tukey–Kramer test, *p < 0.05, **p < 0.01, ns: not significant

To assess the effects of periodontitis on cognitive function, we used the Barnes maze test to evaluate spatial learning. In mice affected for 3 months, the mean time to reach the goal and number of errors were worse compared to those of controls, although not significantly (Fig. 6a). Similar effects of periodontitis were found at 5 M, except that between those groups, the effects were statistically significant (Fig. 6b). This, together with higher mean scores in the diseased groups at 5 M than at 3 M (compare Fig. 6, a and b), highlighting the importance of disease duration.

Furthermore, the association between elevated systemic immune status and impaired cognitive function was suggested by the finding that higher serum levels of IL-1β, TNF-α, and IFN-γ were linked to worse performances in the Barnes maze (Fig. 6c).

To better understand the underlying neural mechanisms of cognitive impairment caused by periodontitis, we examined the hippocampus, a region with neurons crucial for spatial cognition. Given the correlation between blood cytokine levels and the Barnes maze results, we investigated whether microglia are affected by periodontitis, as these brain immune cells are known to prune synapses [29]. Quantification of the number of Iba1-positive microglia [30] in the left and right bilateral hippocampus revealed no significant changes in any of the 3 M models, but the microglia numbers were significantly increased in the 5MP and 5MP_PG models (Fig. 6d). These results indicate that periodontitis increases the number of microglia in the hippocampus. Furthermore, examining the morphological characteristics of these microglia in the 5 M models revealed that the 5MP and 5MP_PG groups exhibited a significant increase in reactive microglia with larger cell bodies and thicker processes [31, 32] compared to that exhibited by 5M_Ctrl (Fig. 6e, f). However, the number of amoeboid microglia [31, 32], which have phagocytic activity and are easily induced by strong inflammation, was not significantly higher in either the 5 M or 5M_PG groups, although the mean value was higher in the 5M_PG group (Fig. 6f). We interpret this as an indication that the periodontitis-induced systemic inflammation was rather mild. Interestingly, clusters of microglia [31] adhering to each other, which are typically observed with aging [31], were observed in only the 5MP_PG group (Fig. 6g).

We also examined adult neurogenesis, which has been reported in rodents, including mice [33]. Inhibition of adult neurogenesis prevents the formation of new memories [33]. We quantified the number of doublecortin (DCX)-positive cells, a marker of newborn neurons [34], in the bilateral dentate gyrus of the hippocampus. The results showed that at 3 M, neurogenesis was significantly reduced in the 3MP_PG model but not in the 3MP model (Fig. 6h), whereas at 5 M, neurogenesis was significantly reduced in both the 5MP and 5MP_PG models (Fig. 6i), highlighting the impact of disease duration and severity.

Despite the reduction in adult neurogenesis, there was no obvious decrease in overall dentate gyrus volume. We examined the shedding of granule cells in the bilateral dentate gyrus and found no major differences, regardless of whether the disease duration was 3 or 5 months (Supplementary Fig. 4a and b). Although there was no quantitative loss of the hippocampus, the findings suggest that decreased neurogenesis and microglial hyperplasia contribute to cognitive decline.

These results indicate that in our periodontitis models, cognitive dysfunction occurs in addition to bone and muscle disorders and that periodontitis leads to cognitive frailty, a condition characterized by the combination of physical frailty and cognitive dysfunction [2].

### Treatment of periodontitis did not reverse the decrease in bone density

Finally, we investigated whether treatment intervention can restore the bone density loss in the femur, which was particularly vulnerable to periodontitis, induced in our periodontitis model. As previously reported [35], the bilateral second molars of the model mice were ligated with silk threads for 3 months, followed by removal of the threads as a therapeutic intervention. The mice in which periodontitis was induced for 3 months and then reared for an additional 2 months after the silk threads were removed were considered as “treated” and referred as the treatment group (5M_Tx).

**Fig. 7:**
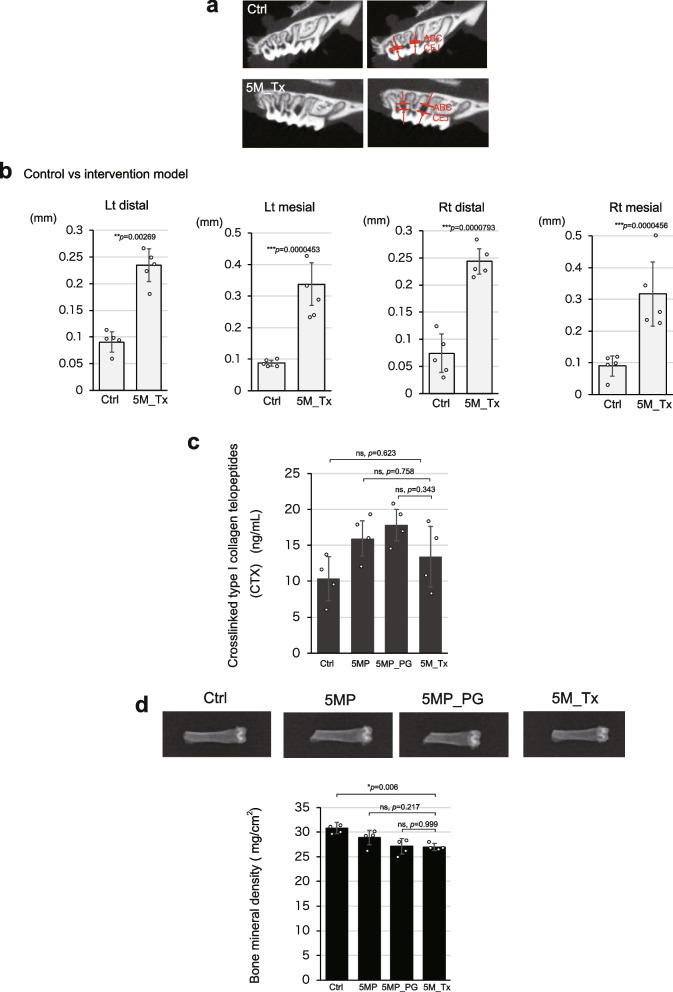
The treatment of periodontitis did not restore bone density. **a** Representative CT images showing bone resorption in the alveolar bone of treated mice (5M_Tx) compared to 5M_Ctrl. **b** Graph quantifying the area of bone resorption in the alveolar bone. The 5M_Tx group did not exhibit recovery of alveolar bone (*n* = 5). **c** Quantification of the bone resorption marker CTX in the 5 M groups (*n* = 4). No worsening of CTX was observed in the 5M_Tx group. **d** Bone density measurements for the 5 M groups (*n* = 4). Bone density was not restored in the 5M_Tx group. Data are presented as mean ± SD. Statistical significance was determined using unpaired two-tailed Student’s *t*-test. One-way ANOVA followed by the Tukey–Kramer test was applied for group analysis, *p < 0.05, ***p < 0.001, ns: not significant

First, we quantified alveolar bone resorption due to periodontitis and found that alveolar bone loss was not restored (Fig. 7a, b). Next, we examined bone resorption markers elevated by periodontitis. The mean value of CTX decreased in the 5M_Tx group compared to the 5MP group and was not as exacerbated as in the 5MP_PG group (Fig. 7c). Similarly, TRACP-5b, which originally showed an increasing trend due to periodontitis (Supplementary Fig. 3b), also showed a decrease in mean values following the intervention. However, these differences were not statistically significant, and the changes in bone resorption markers remained unaffected by the therapeutic intervention.

Moreover, the bone mineral density in the intervention group was not restored. The mean value of 5M_Tx was lower than that of the control group and significantly lower than the control group, indicating that bone density could not be restored (Fig. 7d). These results suggest that periodontitis treatment alone cannot restore femoral bone density once it is lost.

## Discussion

**Fig. 8:**
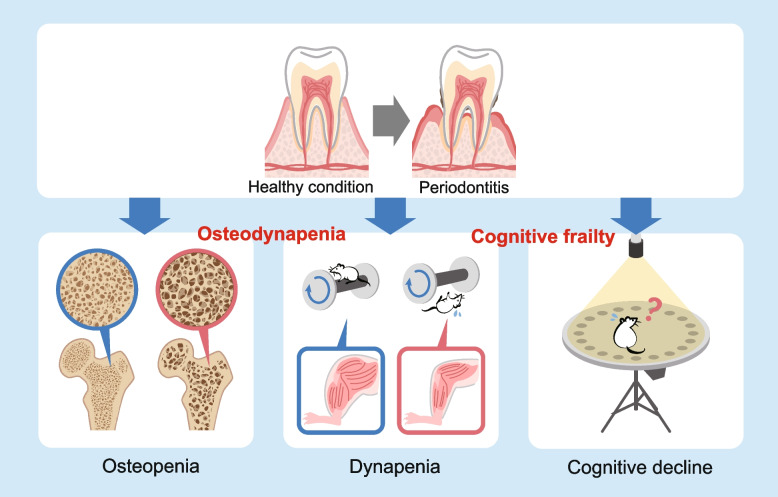
Periodontitis induces osteodynapenia and cognitive frailty. Periodontitis causes osteodynapenia and cognitive frailty by combining osteopenia in the bones, dynapenia in the muscles, and cognitive decline in the brain. The bones were particularly fragile

While examining individual organs in depth is crucial for studying inflammation and aging, it is equally important in geriatric medicine and elderly care to investigate the effects on multiple organs, as they influence each other. The present study shows the comprehensive analysis of multiple organs, revealing that the model mice experienced cognitive frailty, where cognitive functions were impaired alongside the physical frailty, including decreased bone density and muscle weakness (Fig. 8). While previous studies have delved into the effects of periodontitis on a single organ focusing on the inflammatory cascade, this study discovered that periodontitis triggers frailty across multiple organs. This multi-organ frailty presents a high-risk scenario with increased incidence of various diseases and lifestyle dysfunctions, particularly among older patients, underscoring the importance of preventing periodontitis and early therapeutic intervention.

In this study, instead of using the commonly employed intraperitoneal administration of lipopolysaccharide to create inflammation models, we developed a mouse model that closely resembles human periodontitis by ligating silk threads and implanting Pg, known to cause periodontitis. We then examined the relationships among multiple organs to obtain findings relevant to humans. The distinctive feature of this study is our investigation into the interrelationships among multiple organs, aiming to generate insights applicable to human health. Additionally, this study is characterized by its focus on examining how mild, long-term inflammation affects each organ.

Analysis of this mouse model revealed that while the fast-twitch muscles remained unaffected, the slow-twitch muscles were specifically impaired, leading to a condition that may resemble dynapenia. Sarcopenia involves a decrease in muscle mass [3], whereas dynapenia is characterized by a reduction in muscle strength without a loss of muscle mass [5]. In this experiment, the cross-sectional area of the slow-twitch muscles decreased, although muscle weight remained unchanged. This may be due to the thinning of muscle fibers and the accumulation of fat within the muscle. However, fatty tissue was not detectable by staining, representing a limitation of this study. It is possible that fat was lost during the preparation of formalin-fixed paraffin-embedded (FFPE) sections. Additionally, since the soleus muscle in mice, primarily composed of slow-twitch fibers, is very light, the reduction in the muscle fiber area observed could have been obscured by errors in dissection and sampling or by drying. Despite these limitations, scRNA-seq data and the rotarod test clearly indicated significant impairment of the slow-twitch muscles. This highlights the possibility that the observed condition might represent a stage of sarcopenia that has progressed slightly beyond dynapenia.

In contrast, it is generally reported that fast-twitch muscles are primarily affected in sarcopenia [24, 25]. However, in the periodontitis model, slow-twitch muscles were predominantly impacted, suggesting that dynapenia may still initially be caused by periodontitis. Dynapenia is considered a precursor to sarcopenia and could lead to sarcopenia if the disease duration is longer and more severe than that in the present study model. Therefore, older patients with cognitive decline, who often neglect oral care [36], may be in a pre-sarcopenia state.

In addition, in our mouse model, mice with a prolonged disease period exhibited both cognitive dysfunction and dynapenia. This raises the possibility that, if this mouse model is maintained for a longer period, the coexistence of sarcopenia and dementia may be observed. This suggests that prolonged periodontitis could contribute to the progression from dynapenia to sarcopenia, particularly in individuals with cognitive decline who neglect oral hygiene. Future studies should investigate the more long-term effects of periodontitis on muscle health and cognitive function to better understand the link between oral health, dynapenia, and sarcopenia.

However, the decline in physical functions could also be attributed to reduced motivation for movement caused by cognitive impairment. It is hypothesized that mental immobility may lead to a quantitative reduction in slow-twitch muscle fibers. Inflammation caused by periodontitis is anatomically closer to the brain than to the muscles of the lower limbs, making it more likely to affect the brain. Additionally, changes in physical functions and slow-twitch muscles could be interpreted as secondary effects. Additionally, a limitation of this study is the lack of cardiopulmonary function analysis aside from the rotarod test, which prevents us from accounting for the potential influence of cardiopulmonary function.

The femur was found to be particularly vulnerable to periodontitis. Even with mild periodontitis and a short disease duration, the mice exhibited osteopenia, a condition characterized by bone density loss [26]. They also might develop osteodynapenia, which involves both bone density loss and muscle weakness.

Osteodynapenia, marked by reduced muscle strength and bone mass, significantly impacts the quality of life and independence of older patients. When such patients are hospitalized with fractures, multi-organ frailty often develops. This condition may progress to osteosarcopenia [37], underscoring the importance of early intervention and management in elderly care settings to prevent further deterioration.

This finding that bone is more susceptible to impairment than cognitive function is of great importance in geriatric care. While the relationship between chronic inflammation and cognitive function has been documented in clinical studies, it was not previously known that bone could be impaired at an earlier stage. Cognitive function is often treated with relative caution, whereas bone evaluation tends to be an afterthought in general practice. The results of this study suggest that early evaluation of bone density is essential.

A reported molecular mechanism of periodontitis-induced osteopenia involves oncostatin M upregulating the expression of RANKL [38]. Additionally, since insulin and IGF-1 signaling associated with diabetes has been reported to be involved in the molecular mechanism of osteosarcopenia [39], it is possible that similar signaling is also involved in osteodynapenia. Therefore, future research should examine the effects of periodontitis on blood glucose levels, as well as its impact on skeletal muscle and bone health.

We also examined the extent to which osteopenia could be reversed by treating periodontitis. The intervention, as previously reported, involved removing the silk threads that caused the biofilm and then allowing a recovery period. This treatment improved bone resorption markers to some extent but did not restore bone density. Since CTX was the main marker of bone resorption [27, 28], it is suggested that osteoclast activity was reduced or that the exacerbation of osteoclasts was inhibited, potentially halting the progression of bone loss. However, it did not seem to initiate a mechanism to restore the destroyed bone matrix. Once bone density is lost due to periodontitis, aggressive treatment, such as medication in addition to periodontitis treatment, may be necessary to restore bone density.

Periodontitis also significantly impacted cognitive function. This study suggested that, beyond systemic inflammation, the proliferation of microglia in the brain parenchyma is a key cause of cognitive brain damage. Not only the number of microglia but also the number of reactive microglia [31] increased due to periodontitis. The fact that the proportion of reactive microglia to the total microglia has also increased suggests that not only the number but also the nature of the microglial population is changing. Amoeboid microglia [31, 32] are easily induced by severe inflammation, but since the model mice in this study experienced mild inflammation, reactive microglia possibly increased more readily. Moreover, since microglia also play a role in synaptic pruning [29], it is possible that an abnormal increase in microglia leads to the disappearance of synapses.

Additionally, microglial clusters [31] were observed in the 5MP_PG group. Microglial clusters appear in the human brain with aging [31], suggesting that periodontitis induces inflammaging in the brain.

Adult neurogenesis was also reduced by periodontitis. Previous studies have shown that stopping adult neurogenesis with anticancer drugs can cause cognitive decline [33], highlighting the importance of newly born neurons in forming new memories. However, here, although the number of newly born neurons decreased, there was no clear reduction in the overall volume of the hippocampus. This suggests that while neurogenesis is affected, the loss of already incorporated neurons is less severe, possibly due to the milder degree of inflammation compared to other diseases. With longer-term observation of this mouse model, brain atrophy might be detected. However, as introduced in the Background, there have been reports observing toxic proteins produced by Pg in the autopsied brains of AD patients. Nonetheless, this study did not verify such findings. Whether the toxicity specific to Pg, in addition to inflammation, plays a role remains a subject for future investigation.

Moreover, this study utilized female mice. Although evidence regarding sex differences concerning periodontitis and frailty is still lacking, female mice typically exhibit a higher fat-to-muscle ratio than male mice. While periodontitis induced muscle-specific damage, the lower muscle percentage in female mice suggests that even mild damage could lead to a behavioral phenotype. Sex differences are well-documented in the incidence of neurodegenerative diseases such as Parkinson’s [40] and Alzheimer’s [41], yet it remains unknown and a subject of research whether such differences also exist in cognitive dysfunction stemming from inflammatory processes, including osteopenia.

In summary, this study demonstrates that periodontitis induces multi-organ impairment, leading to combined organ frailty. Notably, the femur was identified as particularly susceptible to the effects of periodontitis. These findings provide foundational scientific evidence, using a mouse model, that the progression of periodontitis can cause multi-faceted organ damage, highlighting the systemic impact of the disease.

## Materials and methods

### Mice

C57BL/6 J female mice, 8 weeks old, were purchased from Japan SLC (Shizuoka, Japan) and maintained under specific pathogen-free conditions in animal facilities certified by Tokyo Dental College (Tokyo, Japan). The mice were housed at 24 ± 2 °C with 50–60% humidity on a 12-h light/dark cycle, with ad libitum access to sterile water and a regular diet (MF; Oriental Yeast Co., Ltd., Tokyo, Japan). Five mice were housed per cage. All animal experiments were conducted in accordance with the Institutional Guidelines on Animal Experimentation at Keio University (Tokyo, Japan) and approved by the Keio University Institutional Animal Care and Use Committee (A2023-003) and the Animal Care Committee of the Tokyo Dental College (220,601, 230,602, and 240,602). All methods were performed in compliance with the ARRIVE guidelines.

### Ligature-induced periodontitis mouse model

Periodontitis was induced by placing a 6–0 silk ligature around the maxillary right and left second molars as previously described, with modification [42, 43]. The mice were anesthetized using a two-step protocol. First, isoflurane was administered at an induction concentration of 5% and maintenance concentration of 2.5–4%. Subsequently, a triple anesthetic combination of Domitor (0.75 mg/kg), Midazolam (4 mg/kg), and Vetorphale (5 mg/kg) was prepared to deliver 0.1 mL per 10 g body weight and injected either subcutaneously or intraperitoneally. This protocol ensured adequate and sustained anesthesia throughout the experimental procedure. Alveolar bone loss was evaluated using micro-computed tomography (micro-CT) (Cosmo Scan FX, Rigaku Corp., Tokyo, Japan) under anesthesia at 3 and 5 months after post-ligation. The scan settings were as follows: field of view, 10 mm; 90 kV/88 mA; and exposure time, 2 min. Multiplanar reconstruction (MPR) images were obtained using an image analysis system (OnDemand3D application; Cybermed Corp., Daejeon, Korea). Cross-sections were generated to match the long axis of the maxillary second molar including the two buccal roots. The alveolar bone crest (ABC) and cementoenamel junction (CEJ; ABC-CEJ) distances were measured in the mesial (interdental area between the first and second molars) and distal (interdental area between the second and third molars) areas of the second molar.

### Bacterial culture and preparation

*Porphyromonas gingivalis* strain TDC 60 was grown anaerobically, as described previously [44]. Density of the microorganism was determined by spectrophotometer at an optical density of 660 nm based on a standard curve constructed using colony-forming units (CFU). Pg was transferred to centrifuge tubes and centrifuged at 12,000 × g for 20 min at 4 °C. The supernatants were discarded, and the bacteria were suspended in 2% carboxymethylcellulose in phosphate-buffered saline (PBS) at a concentration of 1 × 10^9 CFU/100 μl. The suspension was immediately placed on ice until its administration to the mice.

### Micro-CT scanning

To evaluate alveolar bone loss due to ligature-induced periodontitis, micro-CT scans were taken using Cosmo Scan FX (Rigaku Corp., Tokyo, Japan) under anesthesia at 3 and 5 months after ligation. The following scan settings: field of view = 10 mm, 90 kV/88 mA, and exposure time 2 min. An MPR image was created using the assistance of the image analysis system (OnDemand3D application, Cybermed Corp., Daejeon, Korea), and a cross section was created that matched the long axis of the second molar and included the two buccal roots. The distances from CEJ to ABC in the mesial area (interdental area between the first and second molars) and distal area (interdental area between the second and third molars) of the second molar were measured.

### Barns maze test

Mice were placed on a round table with 19 pseudoholes and one target hole. The four sides of the table were different landscapes with different shapes to provide a spatial index. After a 5-day adaptation period, we measured the time it took for mice to enter the target hole and the number of errors (number of pseudohole visits) after 3 days had passed.

### Accelerating rotarod test

The rotarod unit (Rota-Rod for Mice RTR-M5; MELQUEST, Japan) was equipped with a rotating rod and 5 separated compartments to accommodate mice. Initially, the rod rotated at 2 rpm. Subsequently, the speed was automatically increased at a constant rate from 2 to 30 rpm over 300 s. The time was recorded when the mouse fell off.

### Single-cell RNA sequencing

Gastrocnemius muscle tissue fixation was performed using formaldehyde, and tissue dissociation was carried out using gentleMACS (Miltenyi Biotec). After hybridizing two types of probes to the RNA of the fixed samples, ligation was performed to form a single probe. The probe-synthesized samples were mixed to ensure an equal number of cells, and encapsulation was performed in the ChromiumX (10 × Genomics) such that one cell was allocated per barcoded primer bead. Following the hybridization of the probes to the capture regions of the barcoded primer beads, extension was performed to add adapter sequences. The extension products were polymerase chain reaction-amplified using indexed primers, creating an indexed sequencing library. Sequencing analysis was conducted using the NovaSeq X Plus, NovaSeq X Series 10B Reagent Kit, control-software v1.1.0, Real Time Analysis (RTA) v4.6.2, and BCL Convert v4.1.7.

### Single-cell RNA sequencing data preprocessing

After filtering out samples with ≥ 5000 counts or mitochondrial gene counts occupying ≥ 10% as part of quality control, we converted raw count data into log2(RPM + 1).

### Factor analysis

With a combination of previously reported marker genes of either fast or slow twitches (Myh1, Myh2, Myh4, and Myh7) and other similar genes (Myh7b, Myh11, and Myh3), we performed factor analysis adopting a practice we introduced in our previous article 23. Note that the similarities of the genes were calculated based on the Jaccard index values regarding registered gene ontology (GO) terms (Supplementary Fig. 1a) applying an algorithm to find similar genes that we implemented in our previous study [45]. Confirming the sampling adequacies (Supplementary Fig. 1b), the parallel analysis suggested the existence of two factors (Supplementary Fig. 1c). We adopted the quartimin rotation and the factor loadings indicated that factor 1 corresponded to fast twitch and factor 2 to slow twitch (Supplementary Fig. 1d). We showed the communalities and uniquenesses of the variables for information in Supplementary Fig. 1e.

### Immunostaining

For immunohistochemistry, fixed brains with 4% PFA/PBS were embedded in paraffin, and 7 μm formalin-fixed paraffin-embedded (FFPE) sections were prepared. After deparaffinization and re-hydration of tissue slides, an antigen revealing step was performed with Target Retrieval Solution (Dako: S1700) according to the user manual. The sections were permeabilized in 0.3% Triton-X100/PBS for 30 min at room temperature. After blocking in Blocking one (Nakalai Tesque: 03953–95) for 30 min at room temperature, the sections were incubated at 4 °C overnight with the following antibodies: rabbit anti-Laminin (Sigma-Aldrich: L9393, 1:500), rabbit anti-Iba1 (Fujifilm: 019–19741, 1:500), and goat polyclonal anti-Doublecortin (Santa Cruz Biotechnology, sc-8066; 1:500). After washing with PBS three times, the sections were incubated for 60 min at room temperature with secondary antibodies conjugated with Alexa Fluor 488 (Thermo Fisher Scientific: A-11034, A-11055) or Alexa Fluor 555 (Thermo Fisher Scientific: A-21429, A-21434). Cell nuclei of the sections were counterstained with Hoechst 33,258 (Sigma-Aldrich, B2883; 10 μg/mL). The sections were mounted on glass slides and analyzed with a confocal laser scanning microscope (LSM700, Carl Zeiss) or an all-in-one fluorescence microscope (BZ-700 or BZ-800, Keyence). Cross-sectional areas were measured as previously reported [46].

### Bone densitometry of the right femur

Dual-energy X-ray absorptiometry (DXA) was performed on the right femur of a mouse under the following conditions:

- Equipment: OsteoSys iNSiGHT
- X-ray output: low energy: 60 kV, 0.8 mA, irradiation time 5 s, high energy: 80 kV, 0.8 mA, irradiation time 5 s

The imaging was conducted by Kureha Special Laboratory Co. (Fukushima, Japan) in a single-blind manner, ensuring that the specimens’ status as periodontitis model mice was unknown to the operators.

### Measurement of inflammatory cytokines in mouse serum

The levels of IL-1β, TNF-α, and IFN-γ in mouse serum were quantified using the MILLIPLEX® Mouse Cytokine/Chemokine Magnetic Bead Panel (Millipore®: KMCYTOMAG-70 K-03). These measurements were performed as single-blind measurements by the Fujita Laboratory Science team.

### Measurement of bone metabolic markers

The levels of serum C-terminal telopeptide of type I collagen (CTX) and tartrate-resistant acid phosphatase (TRACP-5b) were quantified using the Rat-Laps® (Immunodiagnostic Systems Ltd.: AC-06F1) and MouseTRAP™ (Immunodiagnostic Systems Ltd.: SB-TR103). These measurements were performed as single-blind measurements by the Fujita Laboratory Science team.

### Statistical and reproducibility

Statistical analysis was performed using SPSS (version 28.0.1.0; Japan IBM, Tokyo, Japan). All data are presented as mean ± standard error or standard deviation. Two-tailed Student’s *t*-test was used for comparison between the two groups. One-way analysis of variance followed by the Tukey–Kramer test was applied for group analysis. Differences were considered significant at *p < 0.05, **p < 0.01, and ***p < 0.001.

## Supplementary Information

Supplementary Material 1.

## Data Availability

All raw data and sequences of scRNA-seq have been deposited to the DNA Data Bank of Japan (DDBJ) with accession number DRA018993. Any additional information required to reanalyze the data reported in this paper is available from the corresponding author (Hideyuki Okano, email: hidokano@keio.jp).

## Abbreviations

*RANKL*: Receptor activator of nuclear factor-κB ligand
*ABC*: Alveolar bone crest
*Aβ*: Amyloid-beta
*AD*: Alzheimer’s disease
*CEJ*: Cementoenamel junction
*CTX*: C-terminal telopeptide of type I collagen
*DCX*: Doublecortin
*DXA*: Dual-energy X-ray absorptiometry
*Pg*: *Porphyromonas gingivalis*
*sc-RNA-seq*: Single-cell RNA sequencing
*TRACP-5b*: Tartrate-resistant acid phosphatase
