# Supplementary material for: Multi-organ frailty is enhanced by periodontitis-induced inflammaging
Source: Inflamm Regen. 2025 Feb 3;45:3. doi: 10.1186/s41232-025-00366-5 (PMC11789345; doi:10.1186/s41232-025-00366-5)
Supplement: Supplementary file 1 — Supplementary Material 1. [file 41232_2025_366_MOESM1_ESM.pdf]

Supplementary Information for
Multi-organ frailty is enhanced by periodontitis-induced inflammaging

Yoshitaka Kase, Satoru Morikawa, Yuji Okano, Tatsuya Hosoi, Takazumi Yasui, Yoko Taki-Miyashita, Mitsutaka Yakabe, Maraku Goto, Kazuyuki Ishihara, Sumito Ogawa, Taneaki Nakagawa*, Hideyuki Okano*

Taneaki Nakagawa and Hideyuki Okano

Email: tane@keio.jp and hidokano@keio.jp

This PDF file includes:

Supplementary Figures 1 to 4

Supplementary Figure 1. Factor analysis of sc-RNAseq

(**a**) Heat map of Jaccard index of gene ontology terms shared between the two genes in the row and column. (**b**) Bar chart of measurements of sampling adequacy (MSA) per gene. Kaiser-Meyer-Olkin (KMO) index of overall variables (the marker genes) was also shown. (**c**) Parallel analysis using randomly permutated null model indicated the dimensionality of the data matrix of the marker genes was two-dimensional. (**d**) Heat map of factor loadings. (**e**) Stacked bar chart of communality and uniqueness values. (**f**) Histograms of factor scores (left: factor 1; right: factor 2) and manually determined thresholds (borderline to classify a sample positive or negative). (**g**) Scatter plot of the whole samples colored by the classification. The sample sizes for each class were also provided. (**h**) Scatter plot of the whole samples stratified by the classification. Plots were also colored by the experimental conditions. (**i**) Sample proportions of each classification within samples of respective experimental conditions. (**j** and **k**) Scatter plot colored by conditions in UMAP embedding (**j**: fast twitch; **k**: slow twitch).

Supplementary Figure 2. Muscle atrophy markers are unchanged at the transcriptional level

(**a** and **b**) Violin plots of gene expression values in fast twitch stratified with experimental conditions (**a**: Fbxo32; **b**: Trim63). Raw data were also overlaid with horizontal jitter.

Supplementary Figure 3. Serum Levels of TRCP-5b in Mice with Periodontitis

(**a**) In mice with 3-month periodontitis, the mean serum levels of TRCP-5b were elevated in the Pg-treated group compared to those in controls, but this difference was not statistically significant. (**b**) For mice with 5-month periodontitis, the mean serum levels of TRCP-5b were also elevated in the Pg-treated group, though again the difference was not significant. Notably, the intervention in the treatment group (5M_Tx) halted further deterioration of the TRCP-5b serum levels.
Data are presented as the mean ± SD. One-way ANOVA followed by the Tukey–Kramer test was applied for group analysis (n = 5), ns: not significant.

Supplementary Figure 4. Number of Granule Cells in the Hippocampus of Mice with Periodontitis

(a and **b)** In model mice with periodontitis for 3 months and 5 months, there was no significant difference in the number of granule cells in the hippocampus compared to that of controls.
Data are presented as the mean ± SD. One-way ANOVA followed by the Tukey–Kramer test was applied for group analysis (n = 3), ns: not significant.

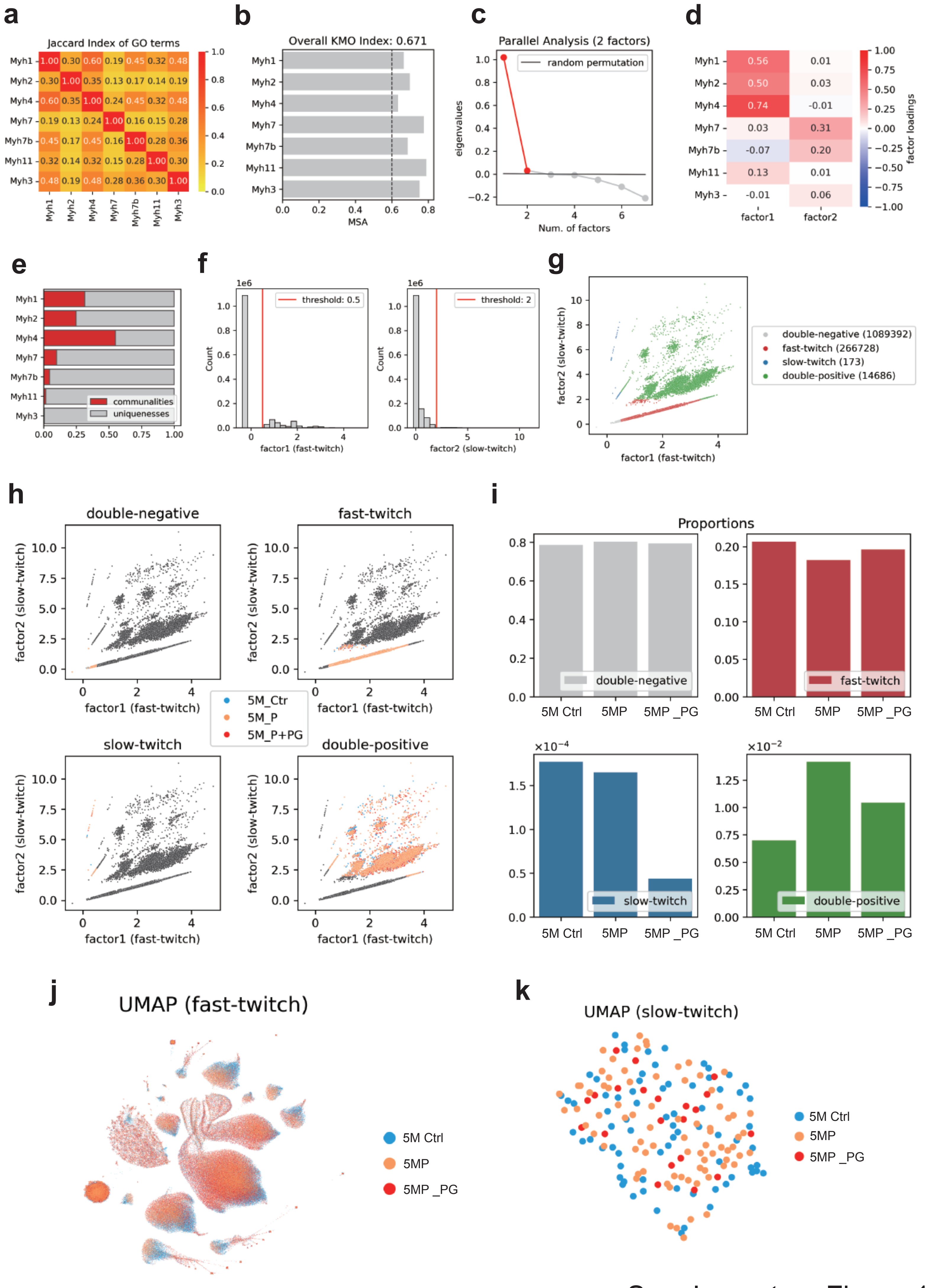

Supplementary Figure 1

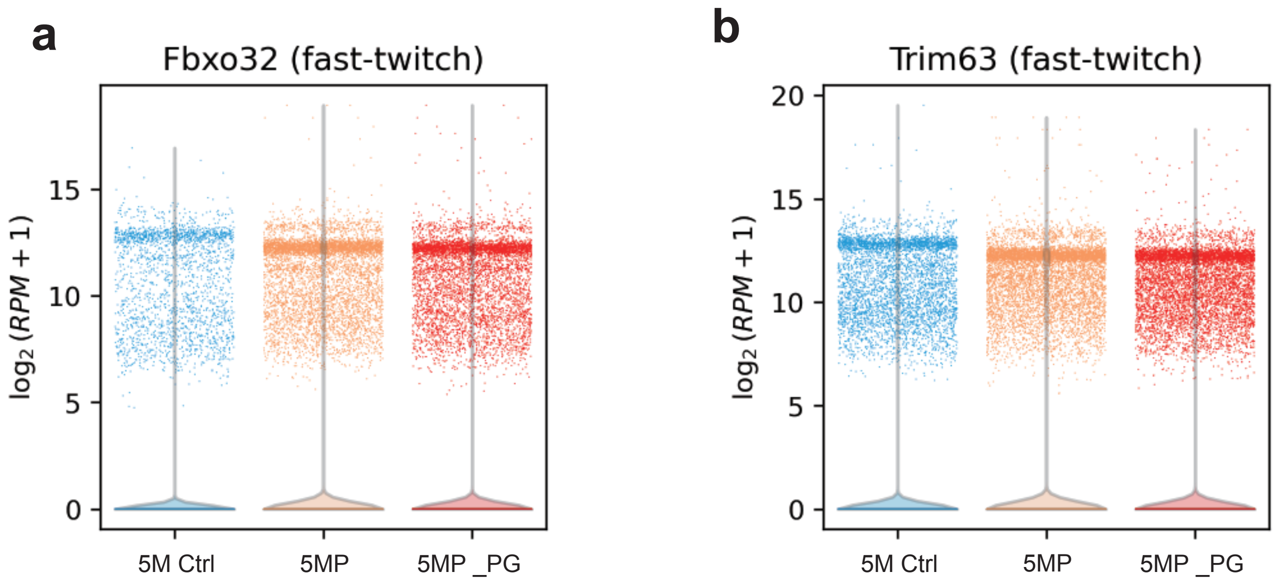

Supplementary Figure 2

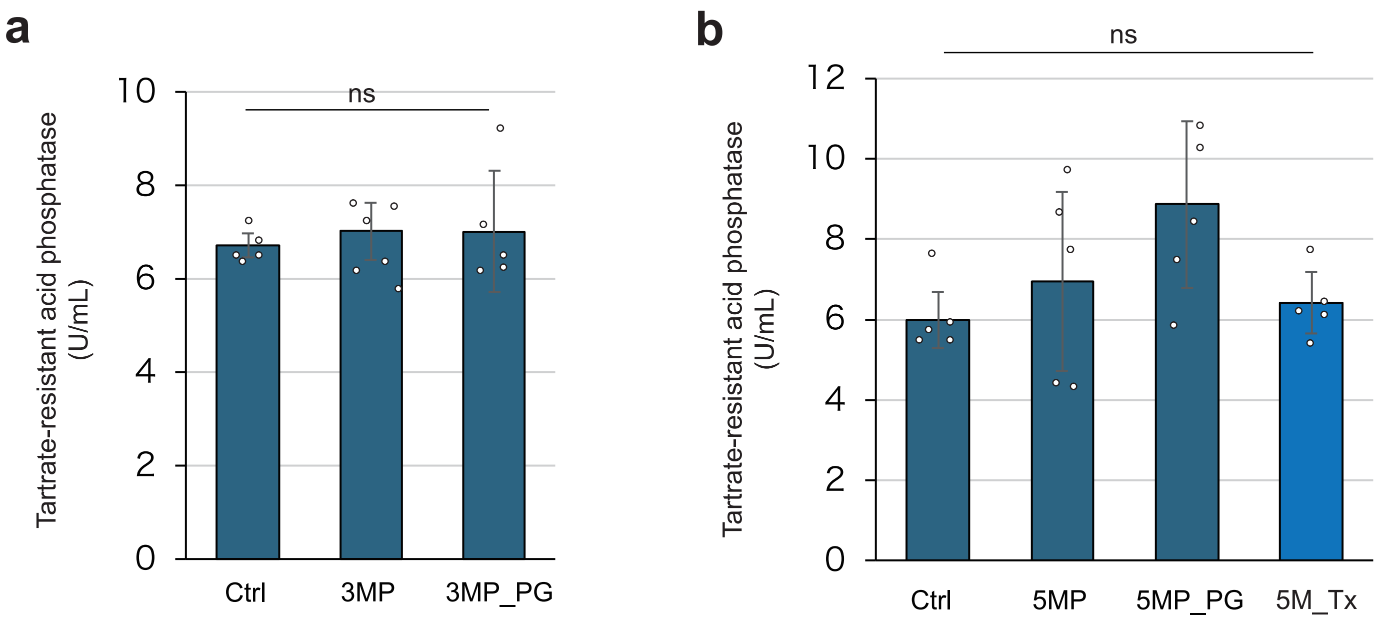

Supplementary Figure 3

a

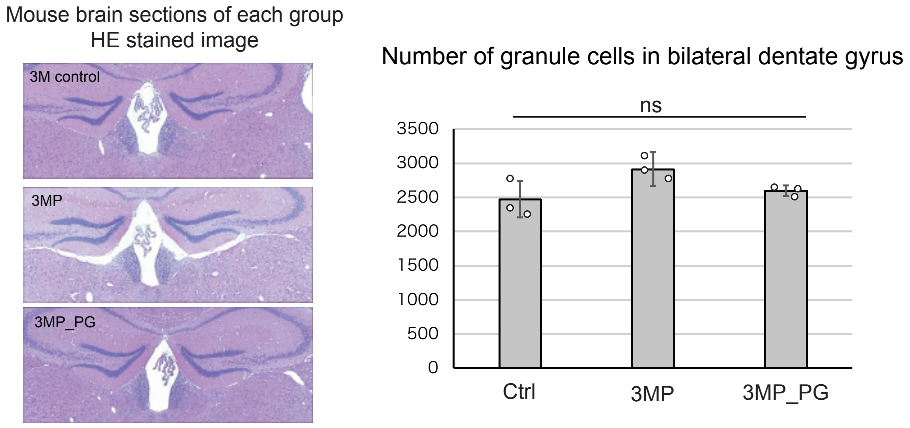

b

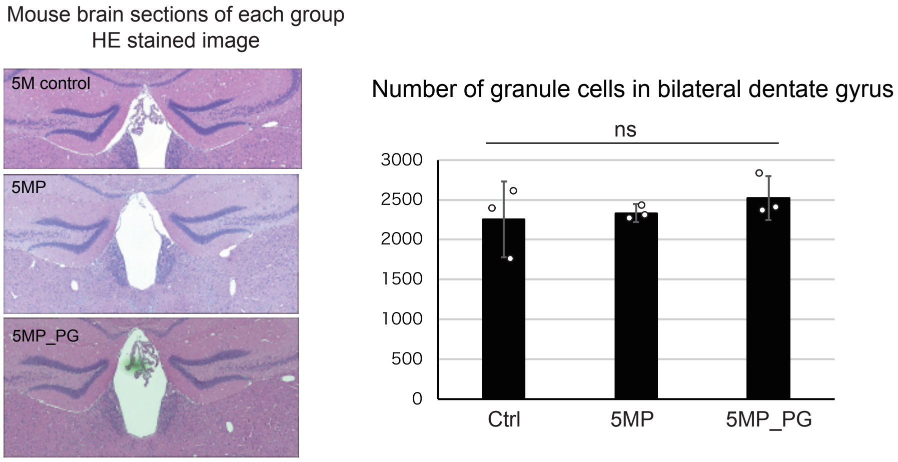

Supplementary Figure 4
